# 1-(4-Meth­oxy­benzyl­idene)-4-methyl­thiosemicarbazide

**DOI:** 10.1107/S1600536810038882

**Published:** 2010-10-09

**Authors:** Yu-Feng Li

**Affiliations:** a Microscale Science Institute, Department of Chemistry and Chemical Engineering, Weifang University, Weifang 261061, People’s Republic of China

## Abstract

The title compound, C_10_H_13_N_3_OS, was prepared by the reaction of 4-meth­oxy­benzaldehyde and 4-methyl­thio­semicarbazide. The dihedral angle between the benzene ring and the thio­urea unit is 8.64 (7)° and an intra­molecular N—H⋯N hydrogen bond generates an *S*(5) ring. In the crystal, inversion dimers linked by pairs of N—H⋯S hydrogen bonds generate *R*_2_^2^(8) loops. The dimers are linked into (001) sheets by further N—H⋯S links.

## Related literature

For background to Schiff bases, see: Casas *et al.* (2000 ▶). For a related structure, see: Li & Jian (2010 ▶).
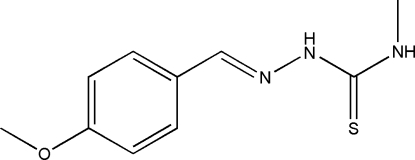


## Experimental

### 

#### Crystal data

C_10_H_13_N_3_OS
*M* *_r_* = 223.29
Orthorhombic, 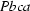

*a* = 13.397 (3) Å
*b* = 9.1271 (18) Å
*c* = 18.799 (4) Å
*V* = 2298.6 (8) Å^3^
*Z* = 8
Mo *K*α radiation
μ = 0.26 mm^−1^
*T* = 293 K
0.25 × 0.22 × 0.18 mm

#### Data collection

Bruker SMART CCD area-detector diffractometer
20742 measured reflections
2627 independent reflections
1746 reflections with *I* > 2σ(*I*)
*R*_int_ = 0.061

#### Refinement

*R*[*F* ^2^ > 2σ(*F* ^2^)] = 0.040
*wR*(*F* ^2^) = 0.146
*S* = 0.94
2627 reflections
136 parameters
H-atom parameters constrained
Δρ_max_ = 0.17 e Å^−3^
Δρ_min_ = −0.29 e Å^−3^

### 

Data collection: *SMART* (Bruker, 1997 ▶); cell refinement: *SAINT* (Bruker, 1997 ▶); data reduction: *SAINT*; program(s) used to solve structure: *SHELXS97* (Sheldrick, 2008 ▶); program(s) used to refine structure: *SHELXL97* (Sheldrick, 2008 ▶); molecular graphics: *SHELXTL* (Sheldrick, 2008 ▶); software used to prepare material for publication: *SHELXTL*.

## Supplementary Material

Crystal structure: contains datablocks global, I. DOI: 10.1107/S1600536810038882/hb5659sup1.cif

Structure factors: contains datablocks I. DOI: 10.1107/S1600536810038882/hb5659Isup2.hkl

Additional supplementary materials:  crystallographic information; 3D view; checkCIF report

## Figures and Tables

**Table 1:** Hydrogen-bond geometry (Å, °)

*D*—H⋯*A*	*D*—H	H⋯*A*	*D*⋯*A*	*D*—H⋯*A*
N1—H1*A*⋯N3	0.86	2.28	2.661 (2)	107
N1—H1*A*⋯S1^i^	0.86	2.86	3.4718 (18)	130
N2—H2*A*⋯S1^ii^	0.86	2.59	3.4359 (17)	169

Symmetry codes: (i) 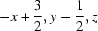; (ii) 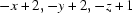.

**Fig. 1.**
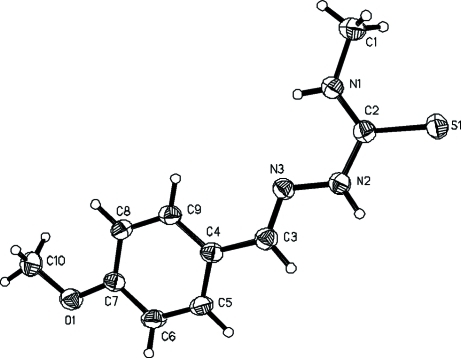
The structure of (I) showing 30% probability displacement ellipsoids.

## supplementary crystallographic information

### Comment

Schiff-base have attracted much attention because they can be utilized as effective ligands to be coordination compounds in coordination chemistry. (Casas *et al.*, 2000). As part of our research for new Schiff-base compounds we synthesized the title compound (I), and describe its structure here. In the molecule structure, the dihedral angle between the benzene ring and the thiourea unit is [8.64 (7)°].

Bond lengths and angles agree with those observed in related compounds (Li & Jian, 2010).

### Experimental

A mixture of 4-methylthiosemicarbazide (0.1 mol) and 4-methoxybenzaldehyde (0.1 mol) was stirred in refluxing ethanol (30 mL) for 2 h to afford the title compound (0.090 mol, yield 90%).  Colourless bars of (I) were obtained by recrystallization from ethanol at room temperature.

### Refinement

H atoms were fixed geometrically and allowed to ride on their attached atoms, with C—H distances=0.97 Å, and with *U*_iso_=1.2–1.5*U*_eq_.

### Crystal data

**Table:**

C_10_H_13_N_3_OS	*D*_x_ = 1.290 Mg m^−^^3^
*M**_r_* = 223.29	Mo *K*α radiation, λ = 0.71073 Å
Orthorhombic, *P**b**c**a*	Cell parameters from 2650 reflections
*a* = 13.397 (3) Å	θ = 3.2–27.2°
*b* = 9.1271 (18) Å	µ = 0.26 mm^−^^1^
*c* = 18.799 (4) Å	*T* = 293 K
*V* = 2298.6 (8) Å^3^	Bar, colorless
*Z* = 8	0.25 × 0.22 × 0.18 mm
*F*(000) = 944	

### Data collection

**Table:**

Bruker SMART CCD area-detector diffractometer	1746 reflections with *I* > 2σ(*I*)
Radiation source: fine-focus sealed tube	*R*_int_ = 0.061
graphite	θ_max_ = 27.5°, θ_min_ = 3.0°
phi and ω scans	*h* = −17→17
20742 measured reflections	*k* = −11→11
2627 independent reflections	*l* = −24→24

### Refinement

**Table:**

Refinement on *F*^2^	Primary atom site location: structure-invariant direct methods
Least-squares matrix: full	Secondary atom site location: difference Fourier map
*R*[*F*^2^ > 2σ(*F*^2^)] = 0.040	Hydrogen site location: inferred from neighbouring sites
*wR*(*F*^2^) = 0.146	H-atom parameters constrained
*S* = 0.94	*w* = 1/[σ^2^(*F*_o_^2^) + (0.1*P*)^2^] where *P* = (*F*_o_^2^ + 2*F*_c_^2^)/3
2627 reflections	(Δ/σ)_max_ = 0.001
136 parameters	Δρ_max_ = 0.17 e Å^−^^3^
0 restraints	Δρ_min_ = −0.29 e Å^−^^3^

### Special details

**Table:**

Geometry. All e.s.d.'s (except the e.s.d. in the dihedral angle between two l.s. planes) are estimated using the full covariance matrix. The cell e.s.d.'s are taken into account individually in the estimation of e.s.d.'s in distances, angles and torsion angles; correlations between e.s.d.'s in cell parameters are only used when they are defined by crystal symmetry. An approximate (isotropic) treatment of cell e.s.d.'s is used for estimating e.s.d.'s involving l.s. planes.
Refinement. Refinement of *F*^2^ against ALL reflections. The weighted *R*-factor *wR* and goodness of fit *S* are based on *F*^2^, conventional *R*-factors *R* are based on *F*, with *F* set to zero for negative *F*^2^. The threshold expression of *F*^2^ > σ(*F*^2^) is used only for calculating *R*-factors(gt) *etc*. and is not relevant to the choice of reflections for refinement. *R*-factors based on *F*^2^ are statistically about twice as large as those based on *F*, and *R*- factors based on ALL data will be even larger.

### Fractional atomic coordinates and isotropic or equivalent isotropic displacement parameters (Å^2^)

**Table:**

	*x*	*y*	*z*	*U*_iso_*/*U*_eq_	
S1	0.89733 (4)	1.04305 (5)	0.58910 (3)	0.0579 (2)	
C7	0.89674 (13)	0.2105 (2)	0.33447 (9)	0.0442 (4)	
N3	0.88845 (12)	0.66880 (17)	0.48831 (8)	0.0467 (4)	
C4	0.92228 (14)	0.4806 (2)	0.40190 (10)	0.0456 (4)	
O1	0.88816 (11)	0.08431 (16)	0.29666 (7)	0.0577 (4)	
N2	0.91161 (12)	0.80887 (17)	0.50939 (9)	0.0509 (4)	
H2A	0.9558	0.8577	0.4862	0.061*	
C8	0.85569 (15)	0.2343 (2)	0.40146 (9)	0.0483 (5)	
H8A	0.8197	0.1606	0.4240	0.058*	
C2	0.86618 (14)	0.8710 (2)	0.56577 (10)	0.0450 (4)	
N1	0.79800 (13)	0.79216 (17)	0.59919 (8)	0.0567 (5)	
H1A	0.7848	0.7061	0.5831	0.068*	
C9	0.86880 (15)	0.3681 (2)	0.43415 (10)	0.0484 (5)	
H9A	0.8412	0.3836	0.4789	0.058*	
C3	0.93581 (15)	0.6236 (2)	0.43363 (10)	0.0505 (5)	
H3B	0.9819	0.6866	0.4129	0.061*	
C5	0.96339 (16)	0.4532 (2)	0.33528 (10)	0.0550 (5)	
H5A	1.0006	0.5259	0.3130	0.066*	
C10	0.83329 (19)	−0.0329 (2)	0.32630 (13)	0.0661 (6)	
H10A	0.8337	−0.1143	0.2940	0.099*	
H10B	0.7657	−0.0023	0.3345	0.099*	
H10C	0.8631	−0.0619	0.3706	0.099*	
C6	0.95017 (17)	0.3214 (2)	0.30188 (10)	0.0584 (5)	
H6A	0.9773	0.3063	0.2569	0.070*	
C1	0.7442 (2)	0.8428 (3)	0.66153 (12)	0.0832 (8)	
H1B	0.6985	0.7681	0.6769	0.125*	
H1C	0.7077	0.9301	0.6499	0.125*	
H1D	0.7907	0.8636	0.6990	0.125*	

### Atomic displacement parameters (Å^2^)

**Table:**

	*U*^11^	*U*^22^	*U*^33^	*U*^12^	*U*^13^	*U*^23^
S1	0.0564 (4)	0.0466 (3)	0.0707 (4)	−0.0056 (2)	0.0113 (2)	−0.0107 (2)
C7	0.0415 (9)	0.0505 (10)	0.0405 (9)	−0.0008 (7)	0.0004 (7)	−0.0047 (8)
N3	0.0450 (9)	0.0478 (9)	0.0473 (8)	−0.0012 (7)	0.0014 (7)	−0.0062 (7)
C4	0.0398 (10)	0.0524 (10)	0.0446 (10)	−0.0010 (8)	0.0011 (8)	−0.0025 (8)
O1	0.0616 (9)	0.0578 (8)	0.0537 (8)	−0.0079 (6)	0.0076 (6)	−0.0105 (7)
N2	0.0492 (10)	0.0480 (9)	0.0557 (9)	−0.0070 (7)	0.0083 (7)	−0.0079 (7)
C8	0.0510 (11)	0.0498 (10)	0.0441 (10)	−0.0016 (9)	0.0055 (8)	0.0060 (8)
C2	0.0393 (9)	0.0470 (10)	0.0488 (10)	0.0024 (8)	−0.0009 (8)	−0.0009 (8)
N1	0.0623 (11)	0.0511 (9)	0.0568 (10)	−0.0110 (8)	0.0156 (8)	−0.0102 (8)
C9	0.0512 (11)	0.0553 (11)	0.0386 (9)	0.0006 (9)	0.0067 (8)	0.0011 (8)
C3	0.0456 (11)	0.0534 (11)	0.0524 (11)	−0.0051 (9)	0.0023 (9)	−0.0020 (9)
C5	0.0572 (12)	0.0587 (12)	0.0492 (10)	−0.0129 (9)	0.0156 (9)	−0.0027 (9)
C10	0.0698 (16)	0.0533 (12)	0.0751 (14)	−0.0098 (10)	0.0038 (12)	−0.0050 (10)
C6	0.0602 (13)	0.0689 (13)	0.0462 (10)	−0.0105 (10)	0.0168 (9)	−0.0102 (9)
C1	0.099 (2)	0.0764 (14)	0.0739 (14)	−0.0251 (14)	0.0394 (15)	−0.0209 (13)

### Geometric parameters (Å, °)

**Table:**

S1—C2	1.683 (2)	C2—N1	1.322 (2)
C7—O1	1.358 (2)	N1—C1	1.451 (3)
C7—C6	1.383 (3)	N1—H1A	0.8600
C7—C8	1.391 (3)	C9—H9A	0.9300
N3—C3	1.277 (2)	C3—H3B	0.9300
N3—N2	1.374 (2)	C5—C6	1.368 (3)
C4—C5	1.391 (3)	C5—H5A	0.9300
C4—C9	1.391 (3)	C10—H10A	0.9600
C4—C3	1.446 (3)	C10—H10B	0.9600
O1—C10	1.413 (2)	C10—H10C	0.9600
N2—C2	1.347 (2)	C6—H6A	0.9300
N2—H2A	0.8600	C1—H1B	0.9600
C8—C9	1.378 (3)	C1—H1C	0.9600
C8—H8A	0.9300	C1—H1D	0.9600
			
O1—C7—C6	115.63 (16)	C4—C9—H9A	119.2
O1—C7—C8	124.96 (17)	N3—C3—C4	124.16 (18)
C6—C7—C8	119.40 (17)	N3—C3—H3B	117.9
C3—N3—N2	114.88 (16)	C4—C3—H3B	117.9
C5—C4—C9	117.63 (17)	C6—C5—C4	121.34 (17)
C5—C4—C3	118.95 (17)	C6—C5—H5A	119.3
C9—C4—C3	123.42 (17)	C4—C5—H5A	119.3
C7—O1—C10	118.68 (15)	O1—C10—H10A	109.5
C2—N2—N3	121.08 (16)	O1—C10—H10B	109.5
C2—N2—H2A	119.5	H10A—C10—H10B	109.5
N3—N2—H2A	119.5	O1—C10—H10C	109.5
C9—C8—C7	119.47 (18)	H10A—C10—H10C	109.5
C9—C8—H8A	120.3	H10B—C10—H10C	109.5
C7—C8—H8A	120.3	C5—C6—C7	120.48 (17)
N1—C2—N2	117.22 (17)	C5—C6—H6A	119.8
N1—C2—S1	123.74 (15)	C7—C6—H6A	119.8
N2—C2—S1	119.04 (15)	N1—C1—H1B	109.5
C2—N1—C1	123.65 (17)	N1—C1—H1C	109.5
C2—N1—H1A	118.2	H1B—C1—H1C	109.5
C1—N1—H1A	118.2	N1—C1—H1D	109.5
C8—C9—C4	121.67 (17)	H1B—C1—H1D	109.5
C8—C9—H9A	119.2	H1C—C1—H1D	109.5

### Hydrogen-bond geometry (Å, °)

**Table:**

*D*—H···*A*	*D*—H	H···*A*	*D*···*A*	*D*—H···*A*
N1—H1A···N3	0.86	2.28	2.661 (2)	107
N1—H1A···S1^i^	0.86	2.86	3.4718 (18)	130
N2—H2A···S1^ii^	0.86	2.59	3.4359 (17)	169

Symmetry codes: (i) −*x*+3/2, *y*−1/2, *z*; (ii) −*x*+2, −*y*+2, −*z*+1.
